# Practical Considerations for the Daratumumab Management in Portuguese Routine Clinical Practice: Recommendations From an Expert Panel of Hematologists

**DOI:** 10.3389/fonc.2021.817762

**Published:** 2022-02-04

**Authors:** Catarina Geraldes, Manuel Neves, Sérgio Chacim, Fernando Leal da Costa

**Affiliations:** ^1^ Departamento de Hematologia Clínica, Centro Hospitalar e Universitário de Coimbra, Coimbra, Portugal; ^2^ Laboratório de Oncobiologia e Hematologia e Clínica Universitária de Hematologia, Faculdade de Medicina, Universidade de Coimbra, Coimbra, Portugal; ^3^ Coimbra Institute for Clinical and Biomedical Research (iCBR), Centro Académico-Clínico de Coimbra (CACC), Coimbra, Portugal; ^4^ Fundação Champalimaud, Lisboa, Portugal; ^5^ Instituto Português de Oncologia do Porto, Porto, Portugal; ^6^ Instituto Português de Oncologia de Lisboa, Lisboa, Portugal

**Keywords:** multiple myeloma, daratumumab, drug-related side effects, adverse reactions, delivery of health care

## Abstract

The recent therapeutic progress in multiple myeloma (MM) has led to the introduction of novel and highly potent drug classes. Daratumumab was the first CD38-targeting antibody showing to be effective and safe in MM patients as monotherapy and in combination regimens, which led to its rapid implementation in clinical practice. Considering that treatment discontinuation for drug-related adverse events can impact patients’ quality of life and outcomes, the treatment decision should consider different factors and be weighted for each patient individually. Here, we aimed to guide clinicians using daratumumab treatment for MM by addressing practical real-world considerations based on an expert panel of Portuguese hematologists. Carefully following the recommendations mentioned in daratumumab’s SmPC, and of those from other drugs used in combination regimens, along with ensuring a good communication with all healthcare professionals involved, is critical to prevent any complications arising from treatment. The risk of infection should be assessed for all patients under treatment with daratumumab and patients should be educated on the potential adverse events. Recommendations on prophylaxis and vaccination should be considered to avoid infections, and delays in the planned therapeutic schedule may be required to prevent adverse consequences of hematological toxicity. Daratumumab treatment is effective and feasible in patients with renal impairment, although careful patient monitoring and a frequent communication with the Nephrology department are of the utmost importance. Sharing clinical practice plays an important role in medical education by allowing to maximize treatment efficacy and minimize its safety risks.

## 1 Introduction

Multiple myeloma (MM) is the second most common hematological malignancy, with over 170 000 new diagnosis per year worldwide (1). MM usually is a symptom-producing cancer causing predominantly bone pain, pathological fractures, fatigue and infections (2). Impressive therapeutic progress has occurred over the last years, with the discovery of several new potential therapeutic targets and the introduction of novel and highly potent classes of anti-myeloma drugs.

Monoclonal antibodies against antigens that are regularly expressed on myeloma cells have been studied intensively for the development of innovative interventions (3). CD38 is a type II transmembrane glycoprotein relevant for the regulation of migration, receptor-mediated adhesion, signaling events and control of intracellular calcium stores (4), and is highly expressed on malignant plasma cells. Daratumumab, the first CD38-targeting antibody, has been shown to be safe and efficacious in patients with MM as monotherapy (5–7) and in combination regimens (8–12). Next to the classic Fc-dependent mechanisms of action, daratumumab also has immunomodulatory effects depleting CD38-positive immune suppressor cells, such as regulatory T cells, regulatory B cells, and myeloid-derived suppressor cells (4, 13–17). Daratumumab has been approved by the European Medicines Agency in 2016 and is currently indicated as monotherapy in patients with relapsed and refractory multiple myeloma, whose prior therapy included a proteasome inhibitor (PI) and an immunomodulatory drug (IMID); and in the following combinations: lenalidomide–dexamethasone [newly diagnosed patients ineligible for autologous stem cell transplant (ASCT) and patients with ≥1 previous therapy], bortezomib–melphalan–prednisone (newly diagnosed patients ineligible for ASCT), bortezomib–thalidomide–dexamethasone (newly diagnosed patients eligible for ASCT), bortezomib–dexamethasone (patients with ≥1 previous therapy), and pomalidomide–dexamethasone (patients with ≥2 previous therapies including lenalidomide and a PI or patients with 1 one prior line of therapy containing a lenalidomide and IP and were lenalidomide refractory) (18). Administration of intravenous daratumumab takes about 7 h for the first infusion and 3–4 h thereafter and was occasionally associated with infusion-related reactions, mainly during the first infusion, in approximately half of patients (18). Long infusion times affect quality of life and strain health-care resources (19). Recently, a subcutaneous formulation of daratumumab (1800 mg daratumumab co-formulated with 2000 U/mL recombinant human hyaluronidase PH20) was developed, with several benefits, including shorter administration time (3–5 min), fewer infusion-related reactions, and simplified drug preparation and administration (19), maintaining its efficacy and safety profile (20–23), not inferior to intravenous administration.

Clinical trials advocate long-term rather than short-term treatment schedules with combinations of several anti–MM drug classes. Although the overall toxicity profile of the recommended combination regimens can be considered favorable, their increasing complexity and prolonged use warrant a heightened vigilance for early and late side effects, mostly because real-life patients are frequently frailer or present with one or more comorbidities. Therefore, the treatment decision process should be weighted for each MM patient individually, considering that early and/or unnecessary tapering or treatment discontinuation for drug-related adverse events may not only reduce patients’ quality of life, but also negatively impact their outcome (24).

In this paper, we aim to guide clinicians using daratumumab treatment for MM, mainly by addressing practical real-world considerations based on experts’ clinical experience, to maximize effectiveness and minimize safety risks.

## 2 Before and After Administration of Daratumumab—What to Ensure and How to Manage Infusion-Related Reactions

The most common adverse events seen with intravenous daratumumab are infusion-related reactions (IRR). From a pooled analysis of two daratumumab trials, IRRs were observed in 48% of the patients and consisted mainly of nasal congestion, cough, allergic rhinitis, throat irritation, chills, vomiting, and nausea (18, 25, 26). Most IRRs occurred during the first infusion and were Grade 1/2, having been safely managed with pre- and post-infusion medications (25). Even though fewer IRRs were experienced with the subcutaneous formulation, their frequency was common following the first injection (with an incidence of 10% in clinical studies). In this topic, we address general pre- and post-infusion recommendations, based on our experience and on the drug’s SmPC, and practical mitigation strategies for IRRs.

### 2.1 What to Ensure Before Initiating Treatment With Daratumumab?

First, considering that daratumumab binds to CD38 on erythrocytes and may interfere with compatibility testing leading to a false positive result in indirect Coombs test (27), it is recommended to notify the Blood transfusion Department that the patient is going to start treatment. Additionally, according to the drug’s SmPC, Erythrocyte phenotyping should be requested prior to starting daratumumab treatment.

We highlight the importance of ensuring adequate training of the nursing team responsible for the administration. There should be education on the administration process, pre-administration drugs, administration route, administration sites, most frequent adverse reactions characteristics, and patient monitoring. Early recognition of IRR and prompt treatment is crucial.

All patients must be provided with a daratumumab patient alert card (18, 27) and informed about the most frequent treatment-emergent adverse events, and should also be warned to immediately contact the medical/nurse team if those occur.

Finally, antiviral prophylaxis should be given, as well as the recommended vaccination (questions detailed on topic B).

### 2.2 What to Ensure for the Administration of Subcutaneous Daratumumab?

Prophylactic therapy should be given 1 hour before the administration of daratumumab (avoid exceeding 3 hours; if given earlier, its effect is not guaranteed throughout the administration). The prophylactic, preferably orally, medication should include in monotherapy or combination therapy, corticosteroid, antipyretics, and H1 antihistamines (18).

The recommended subcutaneous daratumumab dose is 1,800 mg, injected into the subcutaneous tissue of the abdomen, approximately 7.5 cm to the right or left of the navel, for about 3-5 minutes (18). The injection sites should be rotated during the successive injections, and the drug should not be injected in areas where the skin is red, bruised, hard, or showing any scars.

If the patient experiences pain during administration, the delivery rate should be paused or slowed down, and, if pain is not alleviated, a second injection site may be chosen to administer the remaining dose.

### 2.3 What to Ensure After the Administration of Subcutaneous Daratumumab?

IRRs should be monitored during and in the hours following daratumumab administration. After the first administration, it is recommended that the patient remains under medical surveillance within 3 to 4 hours after administration, given the median time to IRRs onset of 3.7 hours (18).

If an anaphylactic reaction or life-threatening (Grade 4) reactions occur, appropriate emergency care should be initiated immediately, and treatment with daratumumab should be immediately interrupted and permanently discontinued.

Post-infusion oral corticosteroids must be given to prevent delayed IRRs, according to the recommendations described in the drug’s SmPC (18). If no major IRRs are experienced after the first three injections, post-injection corticosteroids (excluding background regimen) can be discontinued.

As chronic obstructive pulmonary disease is a known risk factor for hypersensitivity reaction (26), patients with a history of the disease may require additional post-infusion medications to manage respiratory complications (e.g. short- and long-acting bronchodilators and inhaled corticosteroids) (18).

### 2.4 What to Consider in the Assessment of Response to Daratumumab Therapy?

Daratumumab is a human IgG kappa monoclonal antibody that can be detected on serum protein electrophoresis (SPE) and immunofixation (IFE) assays used for monoclonal protein monitoring (18). This interference may impact the determination of complete response and of disease progression in some patients with IgG kappa myeloma protein. In patients with persistent very good partial response (VGPR), where daratumumab interference is suspected, the use of a validated daratumumab-specific IFE assay should be considered to distinguish this drug from any remaining monoclonal protein in the patient’s serum and to allow a correct response assessment (18).

Daratumumab also interferes in the evaluation of minimal/indetectable residual disease (MRD), since this drug binds to residual MM CD38+ cells, preventing the detection of these cells by anti-CD38 antibodies in conventional flow cytometry (28). Therefore, it is important to notify the cytometry laboratory if evaluation of minimal residual disease is requested for a patient under daratumumab treatment.

Key Messages:

- Carefully follow the recommendations mentioned in the SmPC and ensure good communication with all healthcare professionals involved.
- Educate the patient on the treatment, and potential adverse reactions.

## 3 Management and Prevention of Infections in Myeloma Patients Under Treatment With Daratumumab

The overall risk of infection with daratumumab treatment is around 38%, being upper respiratory tract infections the most common. In studies conducted in relapsed/refractory patients, the combination therapy of daratumumab plus lenalidomide and dexamethasone (DRd), and of daratumumab plus pomalidomide and dexamethasone (DPd), led to the highest rates of Grade 3/4 infections. The same results were reported for DRd in studies including newly diagnosed patients. Pneumonia was the most commonly reported severe infection across daratumumab studies, and treatment discontinuation due to infections occurred in 1-4% of patients (18).

The risk of infection may be associated with neutropenia, hypogammaglobulinemia, and NK cell depletion. The risk of neutropenia during daratumumab treatment depends on whether monotherapy or a combination regimen is used. For patients receiving monotherapy, the overall risk of neutropenia ranged from 14 and 19%. In patients treated with combination regimens, the risk of neutropenia is largely higher, ranging from 28 to 48%, and is highest with DRd (8, 11).

### 3.1 What are the General Recommendations Regarding the Prevention and Management of Infections in Patients Under Treatment With Daratumumab?

All patients under treatment with daratumumab should be assessed for risk of infection and monitored for respiratory symptoms or other complaints suggestive of infection. Furthermore, any clinical, analytical or radiological diagnosis should not be delayed, and appropriate treatment should be considered, with special attention to *Streptococcus pneumoniae*.

Since daratumumab can be used in combination with different drugs, the established recommendations for dose adjustment and administration schedule for each drug should be followed when preventing and managing infections in this context, particularly for regimens associated with higher risk of infection.

#### 3.1.1 Bacterial Infections

A number of studies evaluating the benefit of antibiotic prophylaxis in preventing bacterial infections in myeloma patients under treatment reported conflicting results. A randomized phase III trial has suggested the benefit of levofloxacin in this context, but with an unquantified risk of promoting resistance to quinolones (29). Additionally, a meta-analysis evaluating the impact of prophylactic antibiotics in MM patients described the value of prophylaxis in decreasing the overall incidence of infection as modest and concluded that current evidence does not show a decrease in mortality due to infection in this setting (30). Having this in mind, we recommend that antibiotic prophylaxis should be provided only for patients who had neutropenia (< 500 cells/mm^3^) or an infection in a previous treatment cycle.

The most effective measures for the prevention of bacterial infections include: dose adaptation of therapeutics causing drug-induced neutropenia; wisely use of hematopoietic growth factors (G-CSF); and, if needed, postponement of treatment cycles until hematopoietic recovery.

#### 3.1.2 Viral Infections

The risk of viral infection is superior in patients with a higher number of previous treatments and lower tumor response. Antiviral prophylaxis should be considered to prevent herpes zoster virus reactivation (18).

Hepatitis B virus (HBV) reactivation is a well-recognized complication of immunosuppressive treatments for hematologic cancers (31), and some cases have been reported in patients treated with daratumumab (18). Therefore, patients should be assessed for serological risk of Hepatitis B, and those with evidence of positive serology for HBV should be monitored for clinical and laboratory signs of HBV reactivation during treatment and for at least six months after completion. If reactivation occurs, patients should be treated according to current clinical guidelines (32), and it is important to consider consulting an infectious disease specialist. Additionally, daratumumab treatment must be discontinued and its restart after HBV reactivation has been adequately controlled should be discussed with specialists experienced in managing HBV (18).

There may be a similar risk for Hepatitis C, although it has not been reported in the SmPC. There is a risk of CMV reactivation in MM patients, although it varies considerably among CMV-seropositive patients (31) and universal prophylaxis is not recommended.

#### 3.1.3 Fungal Infections

Few studies have investigated fungal infections in MM patients (33). It is unclear whether there is a role for prophylaxis of Candida fungal infections, although prophylaxis according to local practice is recommended in patients with recurrent candidiasis episodes.

There is uncertainty relative to the need for Pneumocystis pneumonia’ prophylaxis. In patients with a previous history of *Pneumocystis jirovecii* infection, it is prudent to consider prophylaxis with cotrimoxazole.

### 3.2 Is Immunoglobulin Replacement Recommended?

Normal human Immunoglobulin (IgHN) is a scarce product, therefore its use must be weighted (34), and only used in situations of controlled disease, hypogammaglobulinemia and severe recurrent infections that led to patient hospitalization (34).

We recommend polyspecific Ig replacement, ideally subcutaneously, in patients with IgG below 500 mg/dL, especially if they have a history of 2 or more episodes of respiratory infection in one year.

### 3.3 What Are the Recommendations Regarding Vaccination in Patients Treated With Daratumumab?

MM patients treated with daratumumab should be vaccinated with prevenar 13, pneumo 23, seasonal influenza vaccine and *Haemophilus influenzae* vaccine, as a recent study showed that there is a similar response compared to the response of patients not treated with daratumumab (35). If patients are not already vaccinated against *Streptococcus pneumoniae*, they should receive this vaccine before starting treatment. The recommended schedule is to start with the 13-valent vaccine followed by the 23-valent vaccine two or more months later (24) and repeat the 23-valent vaccine every 5 years. The vaccine against seasonal influenza should be given annually.

Although patients with MM were not included in the pivotal clinical trials evaluating the efficacy and safety of SARS-CoV-2 vaccines, our advice is to vaccinate as soon as possible, with the following recommendations: delay vaccine administration if severe neutropenia (neutrophils < 0.5 × 10^9^/L); vaccinate between cycles; temporarily discontinue corticosteroids if possible; avoid IV immunoglobulin therapy for 1 month before the first vaccine dose of vaccine and up to 14 days after the second dose (36).

Key Messages:

- Monitor patients under treatment with daratumumab to assess risk of infection.
- Invest in analytical and radiological investigation for patients with suspected infection and in early treatment.
- Carefully follow recommendations on prophylaxis and vaccination.

## 4 Management of hematologic toxicity in multiple myeloma patients under treatment with daratumumab

Haematological toxicities are common in MM and can be a consequence of plasma cell infiltration in the bone marrow, of poorly controlled disease or a result of anti-myeloma therapy (35, 37). As previously described, neutropenia is a very common adverse reaction to daratumumab, along with thrombocytopenia and anemia (18).

### 4.1 What Are the General Recommendations Regarding Hematologic Toxicity in Patients Under Treatment With Daratumumab?

We recommend performing a complete blood count analysis before each treatment cycle. As there is no information on daratumumab’s SmPC regarding lower limits of blood counts for initiating daratumumab treatment, it should be at each physician’s discretion (18). Based on our experience, we consider that daratumumab cycles should be started with neutrophils ≥1 × 10^9^/L; and platelets ≥50 × 10^9^/L (unless if cytopenia is attributed to plasma cell infiltration in the bone marrow).

In the case of haematological toxicity, it is not recommended daratumumab dose reductions, although a delay in drug administration may be required depending on the clinical circumstances and treatment intent (usually in case of grade 4 toxicity or grade 3 thrombocytopenia with bleeding, febrile neutropenia, or any grade neutropenia with infection). Additionally, daratumumab should be interrupted if: neutropenia is inferior to 0.5 × 10^9^/L or if febrile neutropenia is identified, or if thrombocytopenia inferior to 50 × 10^9^/L is present (38). Complete blood count should be monitored weekly (38) and if resolved, treatment with daratumumab may be resumed at the recommended dose without any adjustment (18).

For symptomatic patients and/or those with hemoglobin lower than 8 g/dL, the transfusion of hemoderivatives should be considered according to individual risk, following the best transfusion practices of each institution (38).

Again, when used in combination regimens, the SmPC of the drugs combined with daratumumab should be consulted and the available recommendations for dose adjustment should be followed, as for lenalidomide and bortezomib.

### 4.2 Should Growth Factors Be Used?

Growth factors should be considered if there is bone marrow suppression, to allow starting therapy cycles with neutrophil counts superior to 1 × 10^9^/L (38) as previously described.

Key Messages:

- Even though lower limits of blood counts for initiating daratumumab treatment are not defined, performing a complete blood count analysis before each treatment cycle is recommended.
- Delays in drug administration may be required to prevent adverse consequences of neutropenia and thrombocytopenia.

## 5 Management of Renal Impairment in Daratumumab-Treated Patients

The prevalence of renal impairment in MM at diagnosis is 20 to 40% (39). Renal impairment is associated with a diminish overall survival and higher treatment related toxicity (40). In the trials leading to daratumumab approval (8, 9), patients with severe renal impairment (creatinine clearance of 30 ml/min or less) were excluded. Even though there is no data from clinical trials on patients with severely impaired renal function, some case reports and real-world studies’ results support the efficacy and safety of daratumumab use in patients with severe renal failure, including dialysis patients (41, 42).

### 5.1 What Are the General Recommendations Regarding Renal Impairment in Patients Under Treatment With Daratumumab?

We consider that daratumumab can be used in patients with renal impairment, although we highlight the importance of involving the Nephrology Department when treating such patients. All medical teams involved in the care of these patients should be informed about the concomitant supportive therapies, namely erythropoietin and growth factors.

If the intravenous formulation of daratumumab is used, the infused volume should be reported, usually 1000 mL for first infusion and 500 mL for subsequent administrations. It is important to assure that the Nephrology team is aware of possible signs of infection.

There are no recommended dose reductions in daratumumab treatment, and no clinically relevant differences in drug exposure have been observed between patients with renal impairment and those with normal renal function (18). However, in case of combination therapy, we emphasize the importance of consulting the SmPC of all drugs used and follow the available guidelines regarding dose adjustments, particularly for immunomodulators and alkylating agents.

### 5.2 How Should Patients With Renal Impairment Under Treatment With Daratumumab Be Monitored?

Both daratumumab efficacy and safety results obtained in the clinical trials supporting the drug approval, and the fact that renal impairment has no impact on the drug’s pharmacokinetics and pharmacodynamics (18), constitute advantages for its use in patients with moderate/severe renal failure. However, there should be a close monitoring for the characteristic toxicity of this patient subgroup, and we recommend: assess serum creatinine and complete blood count before each treatment cycle, regardless of patient renal function; during the first treatment cycle, closer monitoring for grade 3/4 hematologic toxicity is recommended in patients with moderate/severe renal function; monitor early signs of infection and immediately start antibiotic therapy if there is any suspected infection; careful monitoring of fluid balance, especially for patients with congestive heart failure.

### 5.3 How Should Daratumumab Be Administered in Dialysis Patients?

Daratumumab should be ideally administered on one day without dialysis session, or as far as possible from the following dialysis session as an alternative. There are no recommendations regarding changes to the mode of administration in the drug’s SmPC.

In our experience, with the new subcutaneous formulation and being able to combine both procedures on the same day (daratumumab administration and dialysis session), dialysis should be done first. If no complications arise from dialysis, then daratumumab subcutaneous administration can be conducted on the same day.

Key Messages:

- Daratumumab treatment is effective and feasible in patients with renal impairment.
- Carefully monitor hematologic toxicity and infections in the first treatment cycles for this set of patients.
- Frequent communication with the Nephrology department is crucial to ensure an appropriate patient follow-up.

## 6 Limitations

Most of the recommendations presented in this manuscript rely on the opinion and clinical experience of a selected group of experts in the disease, meaning they may not be representative of all healthcare institutions in Portugal. Despite this limitation, review of existing data balanced with the experience of this group of experts, highly involved in the management of patients with MM treated with daratumumab, provides relevant information for clinical care.

## 7 Conclusions

The efficacy and tolerability of daratumumab for the treatment of MM have led to a rapid implementation of this drug in monotherapy or in combination with other anti-myeloma agents. A thorough knowledge of daratumumab’s SmPC (and of those from other drugs used in combination regimens) is crucial to prevent any complications arising from treatment and avoid delays in the planned therapeutic schedule.

Proactive patient monitoring and early management of complications is essential to achieve this goal, and the management of these patients should be performed by a multidisciplinary team, whenever necessary.

In this context, sharing clinical practice plays an important role in medical education, particularly for those physicians who are not yet experienced in using daratumumab. Therefore, we aimed to provide practical recommendations on the use of daratumumab treatment for MM, to maximize efficacy and minimize safety risks.

## Data Availability Statement

The original contributions presented in the study are included in the article/supplementary material. Further inquiries can be directed to the corresponding author.

## Author Contributions

All authors equally contributed to project conception, article elaboration and discussion of topics.

## Funding

Unrestricted financial support for medical writing was provided by Janssen-Cilag Farmacêutica Lda. The funder was not involved in the study design; data’s collection, analysis, and interpretation; the writing of this article nor the decision to submit it for publication.

## Conflict of Interest

The authors declare that the research was conducted in the absence of any commercial or financial relationships that could be construed as a potential conflict of interest.

## Publisher’s Note

All claims expressed in this article are solely those of the authors and do not necessarily represent those of their affiliated organizations, or those of the publisher, the editors and the reviewers. Any product that may be evaluated in this article, or claim that may be made by its manufacturer, is not guaranteed or endorsed by the publisher.

## References

[1] Sung H Ferlay J Siegel RL Laversanne M Soerjomataram I Jemal A. Global Cancer Statistics 2020: GLOBOCAN Estimates of Incidence and Mortality Worldwide for 36 Cancers in 185 Countries. CA Cancer J Clin (2021) 71(3):209–49. doi: 10.3322/caac.21660 33538338

[2] Rajkumar SV Dimopoulos MA Palumbo A Blade J Merlini G Mateos MV. International Myeloma Working Group Updated Criteria for the Diagnosis of Multiple Myeloma. Lancet Oncol (2014) 15(12):e538–48. doi: 10.1016/S1470-2045(14)70442-5 25439696

[3] Phipps C Chen Y Gopalakrishnan S Tan D. Daratumumab and Its Potential in the Treatment of Multiple Myeloma: Overview of the Preclinical and Clinical Development. Ther Adv Hematol (2015) 6(3):120–7. doi: 10.1177/2040620715572295 PMC4480519 26137203

[4] van de Donk NW Janmaat ML Mutis T Lammerts van Bueren JJ Ahmadi T Sasser AK. Monoclonal Antibodies Targeting CD38 in Hematological Malignancies and Beyond. Immunol Rev (2016) 270(1):95–112. doi: 10.1111/imr.12389 26864107 PMC4755228

[5] Lokhorst HM Plesner T Laubach JP Nahi H Gimsing P Hansson M. Targeting CD38 With Daratumumab Monotherapy in Multiple Myeloma. N Engl J Med (2015) 373(13):1207–19. doi: 10.1056/NEJMoa1506348 26308596

[6] Usmani SZ Weiss BM Plesner T Bahlis NJ Belch A Lonial S. Clinical Efficacy of Daratumumab Monotherapy in Patients With Heavily Pretreated Relapsed or Refractory Multiple Myeloma. Blood (2016) 128(1):37–44. doi: 10.1182/blood-2016-03-705210 27216216 PMC4937359

[7] Lonial S Weiss BM Usmani SZ Singhal S Chari A Bahlis NJ. Daratumumab Monotherapy in Patients With Treatment-Refractory Multiple Myeloma (SIRIUS): An Open-Label, Randomised, Phase 2 Trial. Lancet (2016) 387(10027):1551–60. doi: 10.1016/S0140-6736(15)01120-4 26778538

[8] Dimopoulos MA Oriol A Nahi H San-Miguel J Bahlis NJ Usmani SZ. Daratumumab, Lenalidomide, and Dexamethasone for Multiple Myeloma. N Engl J Med (2016) 375(14):1319–31. doi: 10.1056/NEJMoa1607751 27705267

[9] Palumbo A Chanan-Khan A Weisel K Nooka AK Masszi T Beksac M. Daratumumab, Bortezomib, and Dexamethasone for Multiple Myeloma. N Engl J Med (2016) 375(8):754–66. doi: 10.1056/NEJMoa1606038 27557302

[10] Mateos MV Dimopoulos MA Cavo M Suzuki K Jakubowiak A Knop S. Daratumumab Plus Bortezomib, Melphalan, and Prednisone for Untreated Myeloma. N Engl J Med (2018) 378(6):518–28. doi: 10.1056/NEJMoa1714678 29231133

[11] Facon T Kumar S Plesner T Orlowski RZ Moreau P Bahlis N. Daratumumab Plus Lenalidomide and Dexamethasone for Untreated Myeloma. N Engl J Med (2019) 380(22):2104–15. doi: 10.1056/NEJMoa1817249 PMC10045721 31141632

[12] Moreau P Attal M Hulin C Arnulf B Belhadj K Benboubker L. Bortezomib, Thalidomide, and Dexamethasone With or Without Daratumumab Before and After Autologous Stem-Cell Transplantation for Newly Diagnosed Multiple Myeloma (CASSIOPEIA): A Randomised, Open-Label, Phase 3 Study. Lancet (2019) 394(10192):29–38. doi: 10.1016/S0140-6736(19)31240-1 31171419

[13] van de Donk NWCJ Richardson PG Malavasi F. CD38 Antibodies in Multiple Myeloma: Back to the Future. Blood (2018) 131(1):13–29. doi: 10.1182/blood-2017-06-740944 29118010

[14] van de Donk NWCJ Usmani SZ. CD38 Antibodies in Multiple Myeloma: Mechanisms of Action and Modes of Resistance. Front Immunol (2018) 9:2134. doi: 10.3389/fimmu.2018.02134 30294326 PMC6158369

[15] Overdijk MB Jansen JH Nederend M Lammerts van Bueren JJ Groen RW Parren PW. The Therapeutic CD38 Monoclonal Antibody Daratumumab Induces Programmed Cell Death *via* Fcγ Receptor-Mediated Cross-Linking. J Immunol (2016) 197(3):807–13. doi: 10.4049/jimmunol.1501351 27316683

[16] Krejcik J Casneuf T Nijhof IS Verbist B Bald J Plesner T. Daratumumab Depletes CD38+ Immune Regulatory Cells, Promotes T-Cell Expansion, and Skews T-Cell Repertoire in Multiple Myeloma. Blood (2016) 128(3):384–94. doi: 10.1182/blood-2015-12-687749 PMC4957162 27222480

[17] van de Donk NWCJ. Immunomodulatory Effects of CD38-Targeting Antibodies. Immunol Lett (2018) 199:16–22. doi: 10.1016/j.imlet.2018.04.005 29702148

[18] European Medicines Agency. DARZALEX Summary of Product Characteristics (2021). Available at: https://www.ema.europa.eu/en/documents/product-information/darzalex-epar-product-information_en.pdf (Accessed June 2021).

[19] Bittner B Richter W Schmidt J. Subcutaneous Administration of Biotherapeutics: An Overview of Current Challenges and Opportunities. BioDrugs (2018) 32(5):425–40. doi: 10.1007/s40259-018-0295-0 PMC6182494 30043229

[20] Usmani SZ Nahi H Mateos MV van de Donk NWCJ Chari A Kaufman JL. Subcutaneous Delivery of Daratumumab in Relapsed or Refractory Multiple Myeloma. Blood (2019) 134(8):668–77. doi: 10.1182/blood.2019000667 PMC6754719 31270103

[21] Clemens PL Xu S Luo M Chari A Usmani SZ Mateos MV. Pharmacokinetics (PK) of Subcutaneous Daratumumab in Patients With Relapsed or Refractory (RR) Multiple Myeloma (MM): Primary Clinical Pharmacology Analysis of the Open-Label, Multicenter, Phase 1b Study (PAVO). Blood (2018) 132(Suppl 1). doi: 10.1182/blood-2018-99-113161

[22] San-Miguel J Usmani SZ Mateos MV van de Donk NWCJ Kaufman JL Moreau P. Subcutaneous Daratumumab in Patients With Relapsed or Refractory Multiple Myeloma: Part 2 of the Open-Label, Multicenter, Dose-Escalation Phase 1b Study (PAVO). Haematologica (2021) 106(6):1725–32. doi: 10.3324/haematol.2019.243790 PMC8168515 32354874

[23] Mateos MV Nahi H Legiec W Grosicki S Vorobyev V Spicka I. Subcutaneous Versus Intravenous Daratumumab in Patients With Relapsed or Refractory Multiple Myeloma (COLUMBA): A Multicentre, Open-Label, Non-Inferiority, Randomised, Phase 3 Trial. Lancet Haematol (2020) 7(5):e370–80. doi: 10.1016/S2352-3026(20)30070-3 32213342

[24] Delforge M Ludwig H. How I Manage the Toxicities of Myeloma Drugs. Blood (2017) 129(17):2359–67. doi: 10.1182/blood-2017-01-725705 28275090

[25] Nooka AK Kastritis E Dimopoulos MA Lonial S. Treatment Options for Relapsed and Refractory Multiple Myeloma. Blood (2015) 125(20):3085–99. doi: 10.1182/blood-2014-11-568923 25838342

[26] McCullough KB Hobbs MA Abeykoon JP Kapoor P. Common Adverse Effects of Novel Therapies for Multiple Myeloma (MM) and Their Management Strategies. Curr Hematol Malig Rep (2018) 13(2):114–24. doi: 10.1007/s11899-018-0443-0 29450683

[27] Tzogani K Penninga E Schougaard Christiansen ML Hovgaard D Sarac SB. EMA Review of Daratumumab for the Treatment of Adult Patients With Multiple Myeloma. Oncologist (2018) 23(5):594–602. doi: 10.1634/theoncologist.2017-0328 29371479 PMC5947446

[28] Perincheri S Torres R Tormey CA Smith BR Rinder HM Siddon AJ. Daratumumab Interferes With Flow Cytometric Evaluation of Multiple Myeloma. Blood (2016) 128(22):5630. doi: 10.1182/blood.V128.22.5630.5630

[29] Drayson MT Bowcock S Planche T Iqbal G Pratt G Yong K. Levofloxacin Prophylaxis in Patients With Newly Diagnosed Myeloma (TEAMM): A Multicentre, Double-Blind, Placebo-Controlled, Randomised, Phase 3 Trial. Lancet Oncol (2019) 20(12):1760–72. doi: 10.1016/S1470-2045(19)30506-6 PMC6891230 31668592

[30] Mohyuddin GR Aziz M McClune B Abdallah AO Qazilbash M. Antibiotic Prophylaxis for Patients With Newly Diagnosed Multiple Myeloma: Systematic Review and Meta-Analysis. Eur J Haematol (2020) 104(5):420–6. doi: 10.1111/ejh.13374 31880853

[31] Teh BW Slavin MA Harrison SJ Worth LJ. Prevention of Viral Infections in Patients With Multiple Myeloma: The Role of Antiviral Prophylaxis and Immunization. Expert Rev Anti Infect Ther (2015) 13(11):1325–36. doi: 10.1586/14787210.2015.1083858 26489539

[32] Myint A Tong MJ Beaven SW. Reactivation of Hepatitis B Virus: A Review of Clinical Guidelines. Clin Liver Dis (Hoboken) (2020) 15(4):162–7. doi: 10.1002/cld.883 PMC7206320 32395244

[33] Liu J Huang H Li Y Liu L Li J Liu Z. Epidemiology and Treatment of Invasive Fungal Diseases in Patients With Multiple Myeloma: Findings From a Multicenter Prospective Study From China. Tumour Biol (2016) 37(6):7893–900. doi: 10.1007/s13277-015-4441-8 26700667

[34] Infarmed. Comissão Nacional de Farmácia e Terapêutica. Recomendação Sobre Utilização De Imunoglobulina Humana Normal (2020). Available at: https://www.infarmed.pt/documents/15786/1816213/Recomenda%C3%A7%C3%A3o+sobre+utiliza%C3%A7%C3%A3o+de+Imunoglobulina+Humana+Normal/27563b3a-71d3-6698-63a3-4074b0b0c0b9 (Accessed June 2021).

[35] Frerichs KA Bosman PWC van Velzen JF Fraaij PLA Koopmans MPG Rimmelzwaan GF. Effect of Daratumumab on Normal Plasma Cells, Polyclonal Immunoglobulin Levels, and Vaccination Responses in Extensively Pre-Treated Multiple Myeloma Patients. Haematologica (2020) 105(6):e302–6. doi: 10.3324/haematol.2019.231860 PMC7271608 31558675

[36] Recomendaciones SEHH. Recomendaciones De Vacunación Covid-19 En El Paciente Hematológico (2021). Available at: https://www.sehh.es/covid-19/vacunacion/124452 (Accessed June 2021).

[37] Chakraborty R Majhail NS. Treatment and Disease-Related Complications in Multiple Myeloma: Implications for Survivorship. Am J Hematol (2020) 95(6):672–90. doi: 10.1002/ajh.25764 PMC7217756 32086970

[38] Basker N. NHS - Chemotherapy Protocol Myeloma - Daratumumab V 1.2 (2018). Available at: https://www.uhs.nhs.uk/Media/SUHTExtranet/Services/Chemotherapy-SOPs/Myeloma/Daratumumab.pdf (Accessed June 2021).

[39] Dimopoulos MA Kastritis E Rosinol L Bladé J Ludwig H. Pathogenesis and Treatment of Renal Failure in Multiple Myeloma. Leukemia (2008) 22(8):1485–93. doi: 10.1038/leu.2008.131 18528426

[40] Gonsalves WI Leung N Rajkumar SV Dispenzieri A Lacy MQ Hayman SR. Improvement in Renal Function and Its Impact on Survival in Patients With Newly Diagnosed Multiple Myeloma. Blood Cancer J (2015) 5(3):e296. doi: 10.1038/bcj.2015.20 25794132 PMC4382661

[41] Kuzume A Tabata R Terao T Tsushima T Miura D Narita K. Safety and Efficacy of Daratumumab in Patients With Multiple Myeloma and Severe Renal Failure. Br J Haematol (2021) 193(4):e33–6. doi: 10.1111/bjh.17412 33748953

[42] Rocchi S Tacchetti P Pantani L Mancuso K Zannetti B Cavo M. Safety and Efficacy of Daratumumab in Dialysis-Dependent Renal Failure Secondary to Multiple Myeloma. Haematologica (2018) 103(6):e277–8. doi: 10.3324/haematol.2018.191122 PMC6058770 29622654

